# Efficient induction of apoptosis by proteasome inhibitor: bortezomib in the human breast cancer cell line MDA-MB-231

**DOI:** 10.1007/s11010-013-1939-5

**Published:** 2014-01-03

**Authors:** Rafał Krętowski, Małgorzata Borzym-Kluczyk, Marzanna Cechowska-Pasko

**Affiliations:** Department of Pharmaceutical Biochemistry, Medical University of Bialystok, Mickiewicza 2A, 15-222 Bialystok, Poland

**Keywords:** Apoptosis, Bortezomib, MDA-MB-231, SA-β-galactosidase, Senescence

## Abstract

The cellular and molecular effects of the proteasome inhibitor—bortezomib—on breast cancer cells are as yet poorly characterised. Bortezomib selectively induces apoptosis in some cancer cells. However, the nature of its selectivity remains unknown. Previously, we demonstrated that: there was no effect of bortezomib action on apoptosis and a time-dependent increase in senescence of human skin fibroblasts. The study presented here provides novel information on cellular effects of bortezomib in breast cancer cells line MDA-MB-231. Our findings demonstrated that in contrast to normal fibroblasts, bortezomib treatment evoked a strong effect on apoptosis in breast cancer cells incubated in hypoxic and normoxic conditions. We observed a time-dependent increase up to 70 % in apoptosis of MDA-MB-231 cells in hypoxic and normoxic conditions. There was no effect of bortezomib action on senescence of these cells. We suggest that bortezomib may be candidates for further evaluation as chemotherapeutic agents for human breast cancer.

## Introduction

The endoplasmic reticulum (ER) is a vital organelle that performs important cellular functions, including the post-translational processing, folding, and trafficking of newly synthesized proteins. A variety of chemicals and adverse environmental conditions, such as hypoxia or nutrient deprivation, can disturb the normal function of this organelle, causing ER stress associated with the accumulation of malfolded protein aggregates [1, 2]. The unfolded protein response (UPR) occurs as an adaptive compensatory reaction to ER stress, characterised by the upregulated synthesis of ER chaperone proteins, the attenuation of protein translation, and the activation of an ER-associated degradation system [2, 3]. In the ER-associated degradation pathway, malfolded or denatured proteins are exported from the ER lumen to the cytosol, and are then degraded by the proteasome [4]. The 26S proteasome is a large multi-subunit complex (2,000 kDa) composed of a central 20S catalytic core particle (20S proteasome) and two 19S subunits that recognize ubiquitinated proteins [5]. Bortezomib (velcade), formerly known as PS-341 is a competitive inhibitor of 20S proteasome activity in whole cells. The chemical name for bortezomib is [(1*R*)-3-methyl-1-({(2*S*)-3-phenyl-2-[(pyrazin-2-ylcarbonyl)amino]propanoyl}amino)-butyl] boronic acid. It is used in the treatment of multiple myeloma and some forms of non-Hodgkin’s lymphoma [5, 6]. Bortezomib inhibition of the proteolytic activity of the 20S proteasome has been shown to induce pro-apoptotic ER stress and inhibit proteasome degradation of nuclear factor-kB (NF-κB) inhibitor in the cancer cell [5, 6].

The inhibition of the proteasome results in many toxic effects, including the accumulation of unfolded and damaged proteins [7–9]. In response to proteasome inhibition, the cell induces specific protective mechanisms, including the unfolded protein response [7], autophagy [10, 11]. However, conditions of excessive ER stress may led to apoptosis [7, 12]. Apoptosis plays a major role in the control of cancer development. In fact, cells encounter multiple apoptotic stimuli during cancer progression, including nutrient deprivation or hypoxia. Accordingly, it has been suggested that the well-documented anti-apoptotic potential of chaperones may play a critical role in the suppression of apoptosis in cancer cells [13, 14]. Recently, attention has shifted towards a novel role of cell senescence in the control of cancer development, and with this shift our view on the role of chaperones in cancer has also evolved towards appreciation of their major role in the regulation of senescence [15].

Senescence can be generally characterised as a cellular stress response. It is a signal transduction process that leads to an irreversible growth arrest of cells in the G1 cell cycle phase [16]. Our knowledge about cell senescence occurring in vivo and, most importantly, as a desired outcome of cancer treatment, is very limited and can still be viewed as an emerging field of study. The term senescence was originally applied to the irreversible growth arrest of cells after prolonged proliferation under in vitro cell culture conditions. Now it has been extended to the irreversible proliferation arrest of cells caused by various stresses, including oxidative damage, telomere dysfunction, DNA damage and oncogene-induced senescence as well [17–19]. Tumour cells are exposed to many different external as well as internal sources of stress; therefore, the induction of senescence constitutes an important block to tumour progression [16]. Senescence is a potent anti-carcinogenic programme and the process of neoplastic transformation involves a series of events that allow cells to bypass senescence by inactivation of senescence-associated pathways. Still, many tumour cells have retained the capacity to senesce in response to external stress stimuli. Most conventional anti-cancer therapies activate DNA damage signalling pathways, thus aiming to induce primarily apoptotic cell death, but often the treated cells do not die by apoptosis but rather undergo growth arrest or senescence. It is not fully understood currently which specific signals cause cells to undergo either senescence or initiate apoptosis [16]. Senescence runs in parallel with an accumulation of damaged proteins in the cell. The attenuation of molecular chaperone inducibility and the simultaneous accumulation of damaged proteins raise the possibility that preservation of protein homoeostasis is a major determinant of the occurrence and duration of cellular senescence [13].

The breast cancer represents a major problem due to both high incidence rate and still unsatisfactory treatment results [20]. The studies were performed on estrogen-independent MDA-MB-231 cells, expressing only β estrogen receptor. Theses cells are highly invasive and metastatic in rodent models [21]. Inhibition of proteasomal degradation system may represent a target in pharmacotherapy of estrogen receptor-negative breast cancer MDA-MB-231 cells. The cellular and molecular effects of the proteasome inhibitor—bortezomib—on breast cancer cells line—MDA-MB-231 are as yet poorly characterised. We decided to study the effect of bortezomib on apoptosis and senescence of breast cancer cells line—MDA-MB-231 incubated in hypoxic and normoxic conditions. We investigated the effect of bortezomib on the activity of senescence marker—SA-β-galactosidase and induction of ORP150 chaperon in cell line—MDA-MB-231 and its correlation with apoptosis of these cells incubated in hypoxic and normoxic conditions.

## Materials and methods

### Reagents

Dulbecco’s modified Eagle’s medium (DMEM) containing glucose at 4.5 mg/ml (25 mM) with Glutamax, penicillin, streptomycin and trypsin–EDTA were provided by Invitrogen (San Diego, USA), passive lysis buffer by Promega (Madison, USA), FBS Gold by Gibco (San Diego, USA), BCA Protein Assay Kit by Thermo Scientific (Rockford, USA), PE Annexin V Apoptosis Detection Kit I by BD Pharmingen™ (CA, USA), Senescence Detection Kit by bioVision (CA, USA), Sigma-Fast BCIP/NBT reagent by Sigma (St Louis, MO, USA), monoclonal (mouse) anti-human ORP150 antibody by IBL (Gunma, Japan), monoclonal (mouse) anti-HIF-1α antibody by BD Biosciences (CA, USA), and alkaline phosphatase-labelled anti-mouse immunoglobulin G by Rockland (PA, USA), precision plus protein standards by Bio-Rad Laboratories (USA).

### Cell cultures

Human breast cancer cell line MDA-MB-231 and human skin fibroblasts (CRL1474) were obtained from American Type Culture Collection (ATCC). Cells were maintained in high-glucose DMEM supplemented with 10% heat-inactivated foetal bovine serum GOLD (FBS GOLD), 2 mM l-glutamine, penicillin (100 U/ml) and streptomycin (100 μg/ml). Cells were cultured in Falcon flasks (BD) in a 5 % CO_2_ incubator (Galaxy S+; New Brunswick), at 37 °C. Subconfluent cultures were detached with 0.05 % trypsin and 0.02 % EDTA in calcium-free phosphate-buffered saline (PBS) and counted in a Scepter cell counter (Millipore).

### Cell viability

Cell viability was measured according to the method of Carmichael [22] using 3-(4,5-dimethylthiazol-2-yl)-2,5-diphenyltetrazolium bromide (MTT). Briefly, cells were seeded in 24-well plate at a density of 100,000 per well. Confluent cells, cultured for 12 h, 24 h and 48 h in normoxic conditions in the concentration from 25 to 1,000 nmol/l, were washed three times with PBS and then incubated with 1 ml of MTT solution (0.25 mg/ml in PBS) for 4 h, at 37 °C in 5 % CO_2_ in an incubator. The medium was removed and 1 ml of 0.1 mol/l HCl in absolute isopropanol was added. Absorbance of converted dye in living cells was measured at wavelength of 570 nm. The viability of MDA-MB-231cells and human skin fibroblasts cultured in hypoxic conditions was calculated as percentage of control cells, incubated in normoxia. All the experiments were done in duplicate in at least three cultures.

### Induction of hypoxia in cell cultures

The cells (5 × 10^5^ in 2 ml of medium) were seeded in six-well plates and incubated until they achieved confluence. The high-glucose DMEM was removed and replaced with 2 ml of the same fresh medium with various concentrations of bortezomib. Control cell cultures were kept in normoxic conditions whereas the test cells were incubated in hypoxic conditions. Hypoxia was evoked by 12 h, 24 h and 48-h incubation of cells in atmosphere containing a reduced to 1 % oxygen concentration in hypoxia chamber (Galaxy 170R; New Brunswick an Eppendorf company). After incubation the culture media were removed, the cell layers were washed with PBS and submitted to the action of lysis buffer for determination of ORP150/GRP170, HIF-1α expression and protein concentration. It allowed the separation of cells and extracellular matrix from the bottom of culture vessels and their suspension in the buffer. The cells incubated on six-well plates were detached with trypsin and analyzed by flow cytometry method.

### Detection of apoptosis

The cells (5 × 10^5^ in 2 ml of medium) were seeded in six-well plates and incubated until they achieved confluence. The MDA-MB-231 cells were incubated in the high-glucose DMEM in hypoxic and normoxic conditions with 25 or 50 nmol/l of bortezomib. The incubation was continued for 12 h, 24 h and 48 h. Apoptosis was evaluated by flow cytometry on FACSCanto II cytometer (Becton Dickinson). The cells were trypsinised and resuspended in DMEM and then in binding buffer. Cells were stained with FITC Annexin V and PI for 15 min at room temperature in the dark following the manufacturer’s instructions (FITC Annnexin V apoptosis detection Kit I). Data were analyzed with FACSDiva software and dead cells were excluded based on forward- and side-scatter parameters.

### Detection of SA-β-galactosidase expression

For cytochemical detection of SA-β-galactosidase staining 5 × 10^5^ cells in 2 ml of growth medium were seeded in Petri dishes (9.5 cm^2^) and incubated for 24 h in the high glucose DMEM. In these conditions they reached 70–80 % confluence. Cells were incubated in the high-glucose DMEM in hypoxic and normoxic conditions with 25 or 50 nmol/l of bortezomib. The incubation was continued for 12 h, 24 h and 48 h. After this time the medium was removed and the cell layers ware washed with 2 ml of PBS. SA-β-galactosidase-positive cells were detected using Senescence Detection Kit (bioVision). Briefly, the cells were fixed with 1 ml of Fixative Solution for 15 min at room temperature and washed with 2 ml of PBS. After washing Staining Solutions Mix was added [940 μl of Staining Solution, 10 μl of Staining Supplement and 10 μl of 20 mg/ml 5-bromo-4-chloro-3-indolyl-β-d-galactopyranoside (X-gal)] at 20 mg/ml concentration, at pH 6.0 which ensures that nonsenescent cells remain unstained. The cells were incubated for 12 h at 37 °C. After incubation the cells were washed with PBS and stained cultures were photographed under an inverted microscope (Olympus CKX 41) at 200-fold magnification. The percentage of SA-β-galactosidase-positive cells was determined by counting the number of blue cells under bright-field illumination, and then the total number of cells in the same field under phase contrast.

### Sodium dodecyl sulphate-polyacrylamide gel electrophoresis (SDS-PAGE)

Cells were washed with cold PBS and solubilised in 100 μl of passive lysis buffer per well. The lysates were centrifuged for 10 min, at 12,000×*g*, at 4 °C. Samples of lysates containing 20 μg of protein were subjected to SDS-PAGE, as described by Laemmli [23]. The Bio-Rad precision plus protein standards were used. Electrophoresis was run for 40–45 min. In each experiment 7.5 % polyacrylamide gel and constant current (25 mA) were used.

### Immunoblotting

The proteins were transferred to nitrocellulose membranes and then pretreated for 2 h with Tris-buffered saline (TBS) containing 0.05 % Tween 20 (TBS-T) and 5 % non-fat dry milk, at room temperature. Membranes were probed for 16 h with a mixture containing monoclonal (mouse) anti-human ORP150 antibody (1: 100) or monoclonal (mouse) anti-human HIF-1α (1: 500) in 5 % dried milk in TBS-T, at 4 °C. Then the alkaline phosphatase conjugated antibody against mouse IgG (whole molecule) was added at a 1:2,500 dilution in TBS-T for 1 h with slow shaking. The nitrocellulose was washed with TBS-T (five times for 5 min) and exposed to Sigma-Fast BCIP/NBT reagent.

### Protein assay

Protein concentration in cell lysates was determined by the method of Smith [24] using BCA Protein Assay Kit (Thermo Scientific, USA). Bovine serum albumin was used as a standard.

### Statistical analysis

Mean values from three independent experiments ± standard deviations (SD) were calculated. Statistical analysis was performed using Student’s *t* test.

## Results

### The effect of bortezomib on viability of MDA-MB-231 cell line

**Fig. 1:**
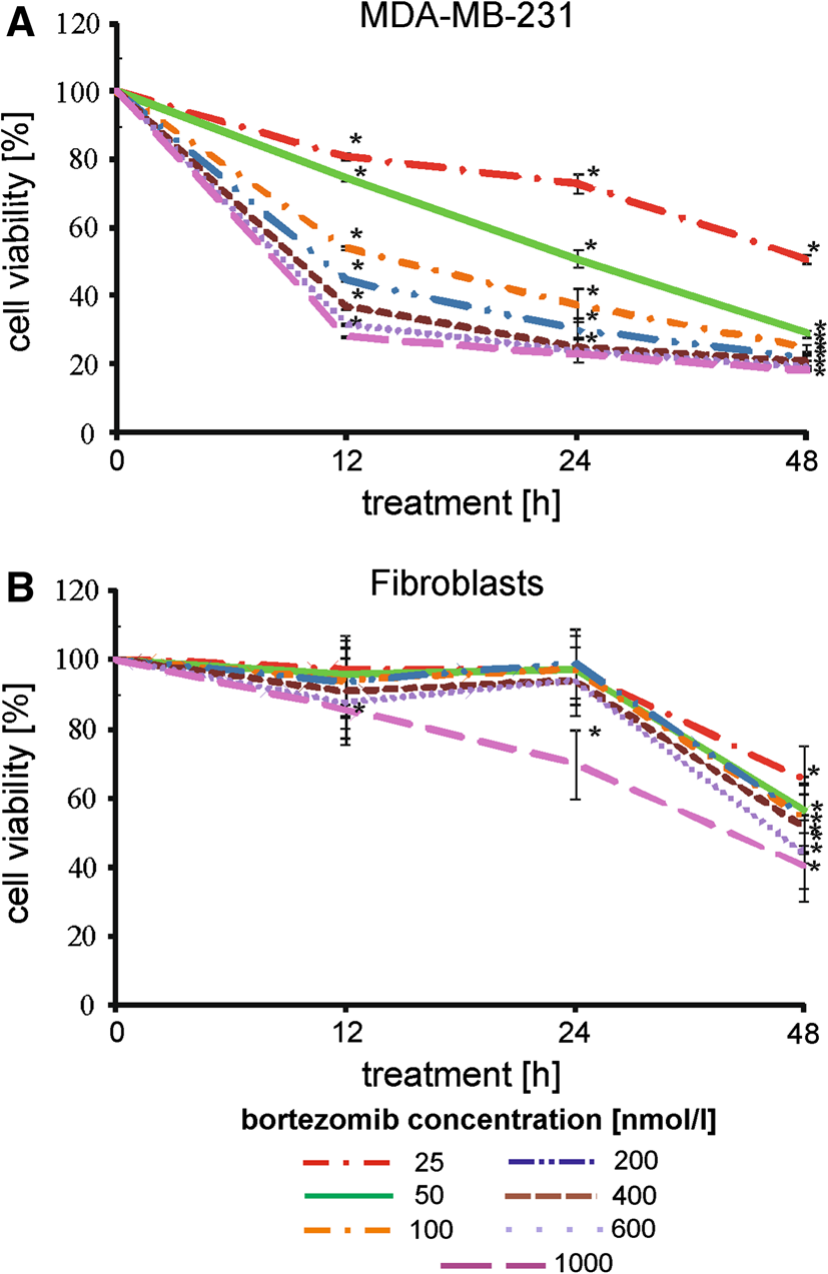
The viability of breast cancer cells treated with different concentrations of bortezomib for 12 h, 24 h and 48 h. The results are mean for pooled triplicate values from three independent experiments. Significant alterations are expressed relative to controls and marked with asterisks. Statistical significance was considered if *p < 0.05

The antiproliferative effect of bortezomib was assessed by MTT method in MDA-MB-231 cells in comparison to normal fibroblasts cultured with increasing concentrations of bortezomib for periods of 12 h, 24 h or 48 h. Figure 1A shows that bortezomib, in the concentration from 25 to 1,000 nmol/l, caused a time-dependent and dose-dependent strong reduction in cell viability of the MDA-MB-231 cells. An evident inhibition in cell viability was observed as early as 12 h. Moreover, IC_50_ was achieved after incubation for 12 h, 24 h and 48 h of MDA-MB-231 cells only with 25 and 50 nmol/l of bortezomib. In cells treated with higher concentrations of bortezomib, the effect on cell viability was markedly more pronounced (Fig. 1A).

In contrast to tumour MDA-MB-231 cells we observed only weak reduction in viability of normal skin fibroblasts using bortezomib in the concentration from 25 to 1,000 nmol/l (Fig. 1B). Moreover, IC_50_ was achieved after 12 h, 24 h and 48 h incubation of fibroblasts with all the tested concentrations of bortezomib (Fig. 1B).

Altogether these results show that bortezomib exhibits a time-dependent and dose-dependent evident inhibition in cell viability of breast cancer MDA-MB 231 cells, in contrast to normal fibroblasts demonstrating high resistance to this inhibitor.

### Detection of HIF-1α in MDA-MB-231 cells submitted to hypoxia

**Fig. 2:**
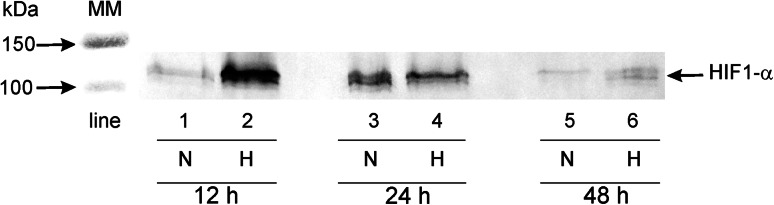
Western immunoblot analysis of HIF-1α synthesized by breast cancer cells has been presented. The cells were incubated in normoxic and hypoxic conditions for 12 h, 24 h and 48 h. Samples containing 30 μg of protein were submitted to electrophoresis and immunoblotting. A representative Western immunoblot is presented. The molecular mass (MM) of the precision plus protein standards are indicated on the left sides of the lane

We also characterised the expression of HIF-1α, a biochemical marker of hypoxia. Figure 2 shows that cells grown in normoxic conditions (for 12 h—lane 1; for 24 h—lane 3; for 48 h—lane 5) did not demonstrate or demonstrate weak expression of HIF-1α. In contrast, those cells incubated in hypoxic conditions demonstrated an intense expression of HIF-1α after 12, 24 and 48 h (Fig. 2, lane 2, 4 and 6). The expression of HIF-1α after 12 h (lane 2) was higher in comparison to 24 h or 48 h (lane 4 and 6).

### The effect of bortezomib on apoptosis of MDA-MB-231 cells

**Fig. 3:**
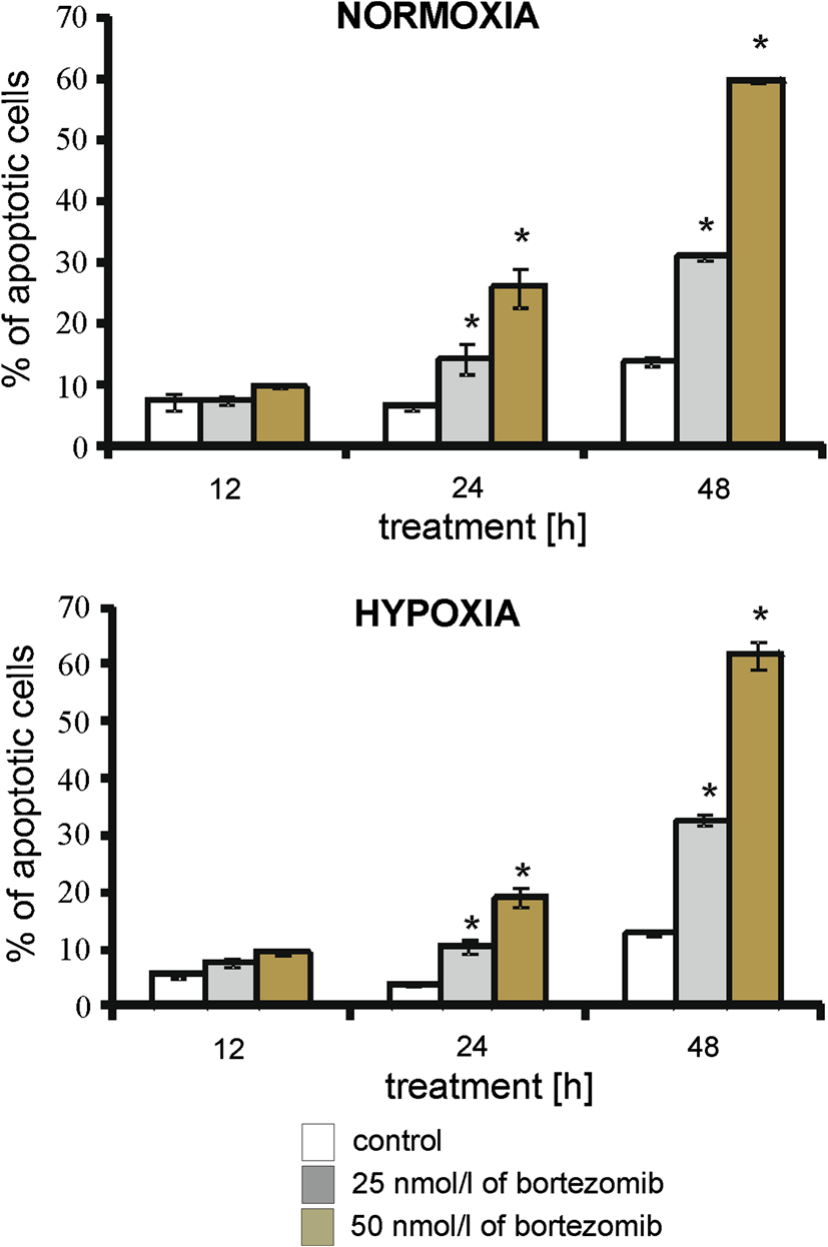
The effect of bortezomib on apoptosis of breast cancer cells. The cells were incubated in normoxic and hypoxic conditions for 12 h, 24 h and 48 h. Mean values from three independent experiments ±SD are presented. Significant alterations are expressed relative to controls and marked with asterisks. Statistical significance was considered if *p < 0.05

Next we investigated whether bortezomib toxicity was due to the induction of apoptosis. Figure 3 shows the per cent of apoptotic MDA-MB-231 cells incubated for 12 h, 24 h and 48 h in normoxic and hypoxic conditions with 25 or 50 nmol/l of bortezomib. Cells incubated for 12 h both in hypoxic and normoxic conditions with 25 or 50 nmol/l of bortezomib did not demonstrate statistically significant differences in comparison to control cells, incubated without bortezomib. In the cells incubated for 24 h and 48 h in hypoxic and normoxic conditions with bortezomib we observed a time-dependent increase in apoptosis of MDA-MB-231 cells (Fig. 3). The per cent of apoptotic cells after 24 h and 48 h of incubation was threefold higher for 25 nmol/l of bortezomib and fourfold higher for 50 nmol/l of bortezomib, in comparison to control cells. Incubation of MDA-MB-231 cells for 48 h with 50 nmol/l of bortezomib resulted in an increase of apoptosis—up to 70%. The per cent of apoptotic cells in the breast cancer cells cultured in hypoxia did not change significantly in comparison to the same cells incubated in normoxia, independently on incubation time and concentration of bortezomib (Fig. 3).

### The effect of bortezomib on SA-β-galactosidase expression

The induction of senescence constitutes an important block to tumour progression. We therefore investigated whether bortezomib induced biomarker of cellular senescence. The SA-β-galactosidase expression was determined with the use of chromogenic X-gal substrate. Normal cells showing traits of division do not undergo reaction with this substrate. However, the cells with evoked cell division blockade become intensely blue stained. Higher SA-β-galactosidase expression in cytoplasm caused the intensification of this reaction.

**Fig. 4:**
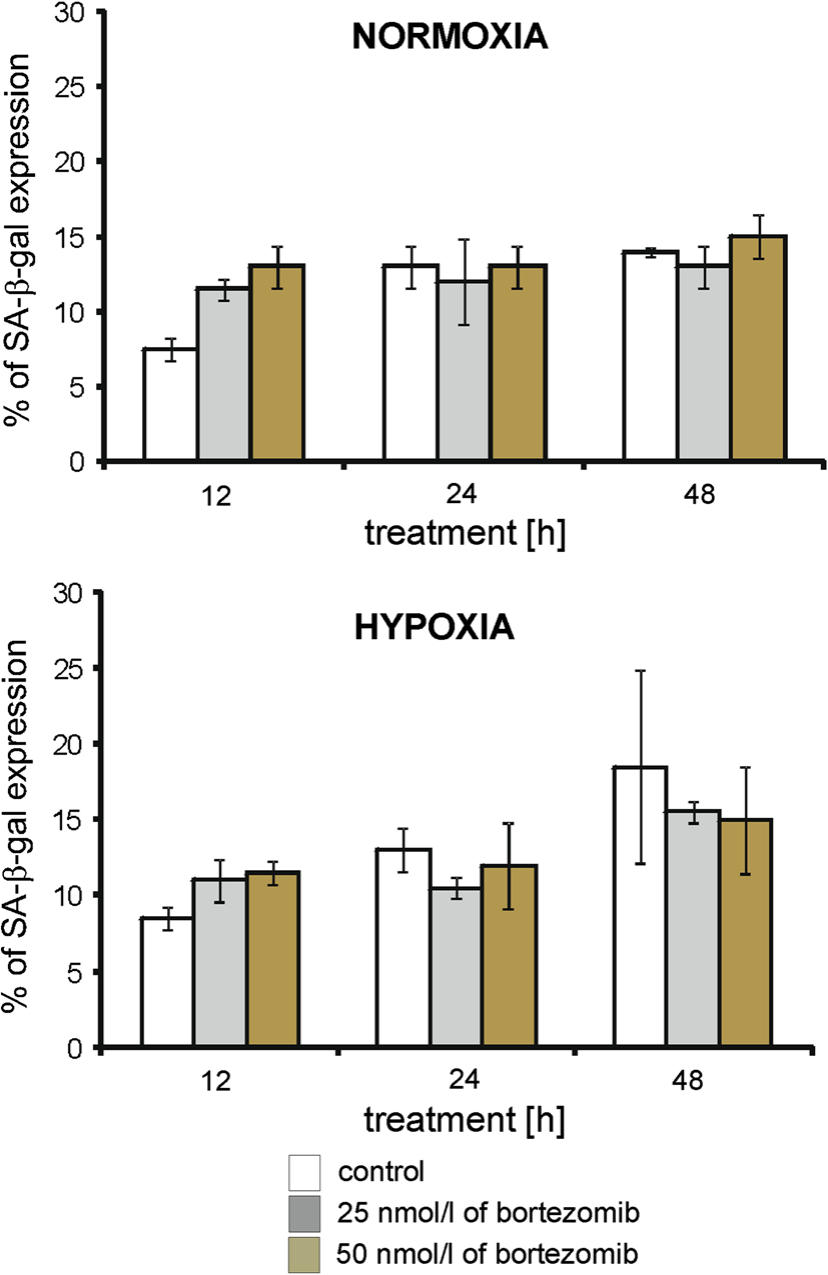
The effect of bortezomib on percentage of SA-β-galactosidase expression in MDA-MB-231 cells. The cells were incubated for 12 h, 24 h and 48 h in normoxic and hypoxic conditions. Mean values from three independent experiments ±SD are presented

Figure 4 shows the per cent of SA-β-galactosidase-positive MDA-MB-231cells incubated for 12 h, 24 h or 48 h in normoxic and hypoxic conditions with 25 or 50 nmol/l of bortezomib. The per cent of SA-β-galactosidase-positive MDA-MB-231 cells did not change significantly both in hypoxia and normoxia (Fig. 4), independently on incubation time and concentration of bortezomib. Moreover, no significant differences between cultures incubated with various concentrations of bortezomib in comparison to control were observed (Fig. 4).

**Fig. 5:**
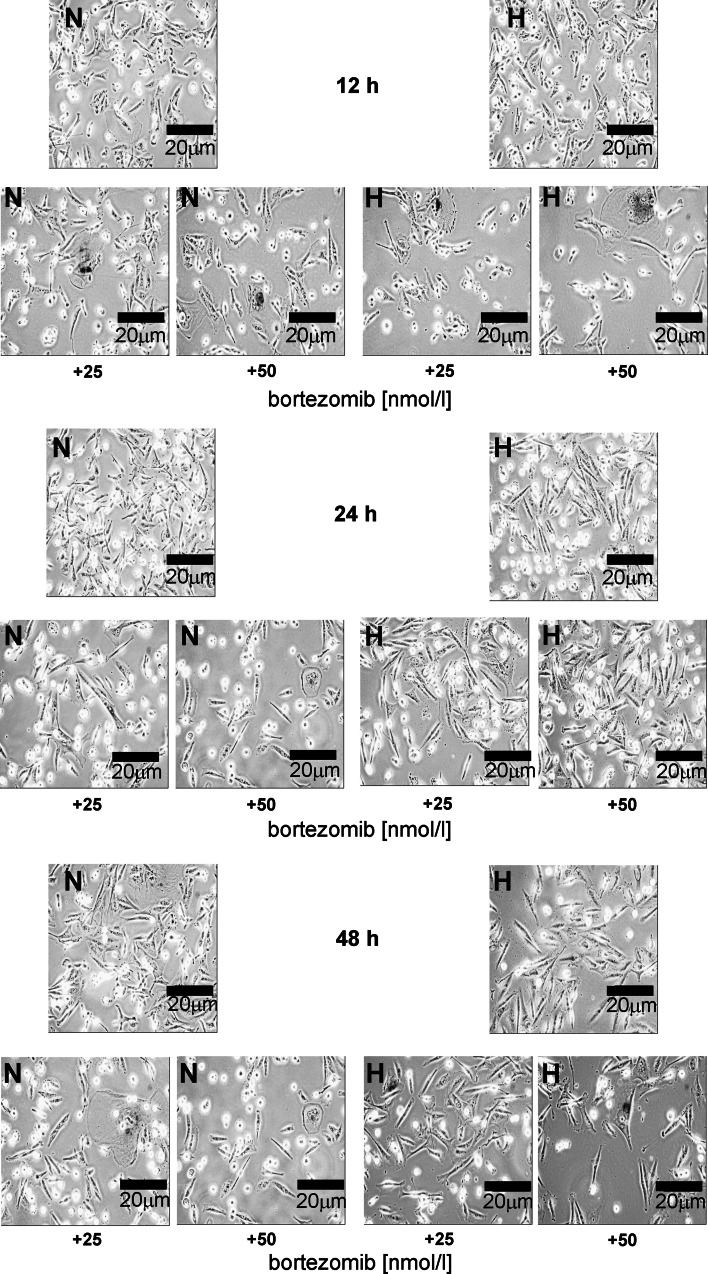
Cytochemical staining for SA-β-galactosidase in MDA-MB-231 cells. The cells were incubated for 12 h, 24 h and 48 h in normoxic and hypoxic conditions. A representative photograph (magnification ×200) is presented

Figure 5 displays a representative photographs of positive for SA-β-galactosidase staining MDA-MB-231 cells incubated for 12 h, 24 h and 48 h in normoxic and hypoxic conditions with 25 or 50 nmol/l of bortezomib. The amount of SA-β-galactosidase-positive MDA-MB-231cells did not change significantly both in hypoxia and normoxia, independently on incubation time and concentration of bortezomib (Fig. 5). Moreover, no significant differences between cultures incubated with 25 or 50 nmol/l of bortezomib in comparison to control were observed (Fig. 5).

### The effect of bortezomib on the expression of ORP150/GRP170

**Fig. 6:**
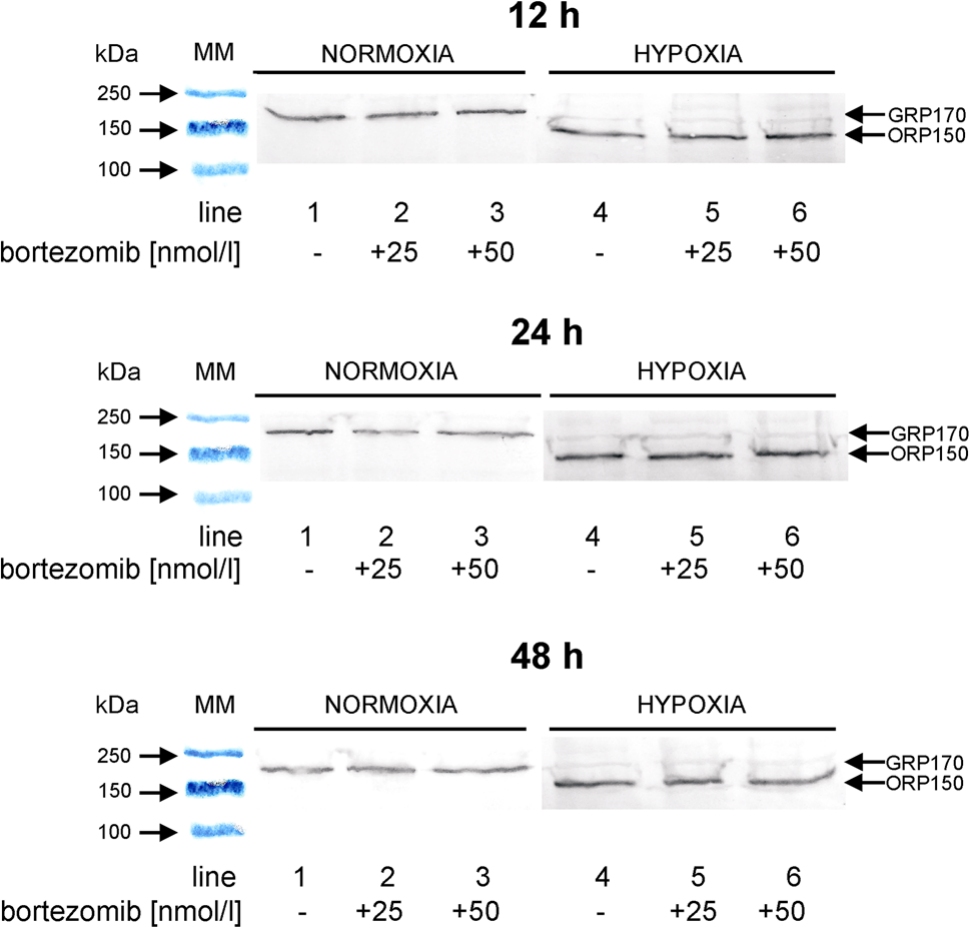
Western immunoblot analysis of ORP150/GRP170 expression in MDA-MB-231cells incubated with various concentrations of bortezomib in normoxic (*lanes 1–3*) and hypoxic (*lanes 4–6*) conditions for 12, 24 and 48 h. *Lanes 1* and *4*—without bortezomib, *lanes 2* and *5*—with 25 nmol/l of bortezomib, *lanes 3* and *6*—with 50 nmol/l of bortezomib. Samples containing 20 μg of protein were submitted to electrophoresis and immunoblotting. A representative Western immunoblot is presented. The molecular mass (MM) of the precision plus protein standards are indicated on the left side of the lanes

Overexpression of Hsp70 chaperons (ORP150 belongs to this family) may protect cancer cells against entering the apoptotic pathway. Figure 6 shows the expression of ORP150, and its glycosylated form GRP170, in MDA-MB-231 incubated in normoxic and hypoxic conditions with 25 and 50 nmol/l of bortezomib. We observed induction of ORP150 expression in hypoxic conditions only.

The expression of GRP170 in MDA-MB-231 was observed in cultures incubated in normoxic conditions for 12 h, 24 h and 48 h, independently on concentration of bortezomib (Fig. 6; lanes 1–3). It is of interest that MDA-MB-231 cells incubated in normoxia did not express ORP150 (Fig. 6; lanes 1–3). The induction of ORP150 expression was observed in hypoxic conditions in MDA-MB-231 incubated for 12 h, independently on concentration of bortezomib (Fig. 6; lanes 4–6). Prolongation of incubation up to 24 h and 48 h resulted in intensification of ORP150 expression. Moreover, a slight expression of GRP170 was observed in MDA-MB-231cells incubated in hypoxic conditions (Fig. 6; lanes 4–6).

## Discussion

The 26S proteasome inhibitor, bortezomib, selectively induces apoptosis in some cancer cells. However, the nature of its selectivity remains unknown. The work presented here provides novel information on cellular effects of bortezomib in MDA-MB-231 breast cancer cell line. We observed that bortezomib reduces cell viability of the tested cultures in a concentration and time-dependent manner. Our results demonstrate the efficient induction of apoptosis by proteasome inhibitor bortezomib in the human MDA-MB-231 breast cancer cell line. We observed a time-dependent increase, up to 70 %, in apoptosis of MDA-MB-231 cells. We demonstrated that the per cent of apoptotic cells in the breast cancer cells cultured in hypoxia did not change significantly in comparison to the same cells incubated in normoxia, independently on incubation time and concentration of bortezomib. There was no effect of bortezomib action on senescence of breast cancer cells. In contrast to breast cancer cells we found that there was no effect of bortezomib action on apoptosis and a time-dependent increase in senescence of normal skin fibroblasts. Our previous study shown that: bortezomib caused a time-dependent increase in senescence of normal fibroblasts, especially of these incubated in hypoxic conditions. The SA-β-galactosidase expression raised over 40 % after 24 h and over 70 % after 48 h of incubation [25].

The maintenance of protein homoeostasis in cell requires the activities of chaperones and the ubiquitin–proteasome system, which together serve to inactivate and degrade misfolded proteins. When proteins are not folded properly, they are directed to 26S proteasomal degradation. If misfolded or unfolded proteins are not degraded by the proteasome, they form aggregates and lead to the ER stress. The ER stress triggers UPR to reduce the accumulation of unfolded proteins and restore the ER function. When protein aggregation or ER stress persists, the UPR signalling switches from the pro-survival to pro-apoptotic. Consequently, the 26S proteasome complex also plays an important role in regulation of the ER stress and cell survival. Therefore, inhibition of the proteasomal function in cancer cells would promote apoptosis and have an anti-tumour function. In fact, the inhibition of the proteolytic activity of the 26S proteasome has been shown to induce pro-apoptotic ER stress in multiple myeloma [26], pancreatic [27], head and neck cancer [7], and non-small cell lung carcinoma [28].

Proteasomal activity is essential for eliminating of excess proteins and, by counteracting protein production, establishing steady protein levels [29]. The ubiquitin–proteasome pathway represents the major pathway for intracellular protein degradation. The 26S proteasome is responsible for the degradation of approximately 80 % of cellular proteins, including misfolded and mutated proteins as well as those involved in the regulation of development, differentiation, cell proliferation, signal transduction, apoptosis and antigen presentation [5]. Prolonged proteasome inhibition induces stress responses that initiate apoptosis via intrinsic pathway [29]. This is exploited clinically in the treatment of multiple myeloma with the proteasome inhibitor bortezomib. Inhibition of proteasome activity by bortezomib is associated with an accumulation and transcriptional induction of BH3-only proteins such as PUMA, BIM, NOXA or BIK. BH3-only proteins antagonise antiapoptotic BCL-2 family members such as BCL-2, BCL-xL or Mcl-1 and can activate the proapoptotic members BAX and BAK [30]. Activated BAX and BAK form pores in the outer mitochondrial membrane, resulting in cytochrome c and activators of caspases (Smac/Diablo) release from the intermembrane space into the cytosol. This results in caspase-9 activation, inhibition of IAP (inhibitor of apoptosis proteins), and subsequent apoptosis execution by effector caspases [30]. Induction of NOXA has been reported to be a key mechanism in bortezomib-mediated apoptosis which is independent of P53 status but dependent on c-Myc [31–34]. Bortezomib-mediated apoptosis is accompanied by induction of c-Jun-NH2 terminal kinase; generation of reactive oxygen species; release of cytochrome c, the second mitochondria-derived activator of caspases and apoptosis-inducing factor; and activation of the intrinsic caspase-9 pathway and extrinsic caspase-8 pathway [5].

In agreement with the cytoprotective role of molecular chaperones it has been shown that they can prevent stress-induced apoptosis [13, 14]. Overexpression of Hsp70 chaperons (ORP150 belongs to this family) prevents cytochrome c release from mitochondria, blocks apoptosome formation by binding to the apoptotic protease-activating factor (Apaf-1), inhibits the release of apoptosis-inducing factor (AIF) from mitochondria and prevents the loss of mitochondrial transmembrane potential. The AIF released from mitochondria binds to Hsp70 and this interaction makes impossible the nuclear import of AIF [15].

Senescence is thought to play an important role in tumour suppression. Cellular senescence is more than just replicative senescence. It is a common programme that is activated by normal cells in response to various types of stress. It has been named ‘stress-induced premature senescence’ [19]. This type of senescence can act as a tumour suppressor in order to prevent damaged cells to multiply as well as a secondary outcome for cancer cells due to therapeutic treatments [16]. Tumour suppressors prevent cells from transforming into cancer by forming molecular barriers for genomic instability and infinite proliferation. They normally induce either apoptosis or a permanent cell cycle arrest—senescence. Unfortunately, which factors determine apoptosis or senescence are not known at present [16].

Chaperones induction in cancer cells can lead to cancer progression and may be a major cause of chemotherapeutics resistance. It is one of the mechanisms protecting cancer cells against entering the apoptotic pathway. Hence, chaperone inhibition may be a promising tool to decrease cytoprotection and to initiate apoptosis of cancer cells [16, 35]. Senescence and apoptosis normally counteract tumour development and cancer cells must, therefore, overcome these important tumour suppressor mechanisms to disrupt this barrier. Senescence emerges as an important tumour suppressor mechanism *in vivo*. Understanding and application of cellular senescence and apoptosis for cancer therapy has recently become a field of extensive research.

In conclusion, bortezomib represents compounds which are able to induce apoptosis in human cancer cells in low dose. We suggest that bortezomib may be candidates for further evaluation as chemotherapeutic agents for human breast cancer.
